# The Intriguing Case of Impacted Teeth 11, 12, and 13: Unveiling the Dental Dilemma

**DOI:** 10.7759/cureus.51611

**Published:** 2024-01-03

**Authors:** Dhwani Suchak, Ranjit Kamble, Jeni Mathew, Rutuja Ragit, Japneet Kaiser, Nishu Agarwal, Ruchika Pandey

**Affiliations:** 1 Orthodontics, Sharad Pawar Dental College, Datta Meghe Institute of Higher Education and Research, Wardha, IND; 2 Pediatric and Preventive Dentistry, Sharad Pawar Dental College, Datta Meghe Institute of Higher Education and Research, Wardha, IND

**Keywords:** comprehensive treatment, supernumerary teeth, surgical exposure, impacted teeth, missing teeth

## Abstract

This case study offers a rare and difficult condition involving the impaction of teeth 11, 12, and 13, providing a severe dental challenge. A thorough examination was performed on the patient, which included clinical evaluations and radiographic examinations. Because the impacted teeth were causing discomfort and functional impairment, a multidisciplinary approach was required, which included surgical exposure followed by traction forces to level and align the impacted teeth.

The abstract emphasizes the case's complexity, digging into the diagnosis process and the establishment of a personalized treatment strategy. The complexities of handling many impacted teeth are explored in length, including surgical intervention, orthodontic considerations, and postoperative care.

## Introduction

The maxillary incisors are important for aesthetics since they are the first teeth to be visible on smiling and during speech in most people. The absence of an incisor can also cause functional issues with speaking, such as difficulty producing the "s" sound. As a result, appropriate tooth eruption, location, and morphology are critical for normal phonation and aesthetics [1].

Pathological obstruction, dental deformity, abnormal position of the tooth bud, non-vital or ankylosed primary teeth, endocrine problems, or bone disorders can all cause maxillary incisor failure to erupt [1]. If the contralateral incisor erupted six months earlier, the lower incisors erupted more than a year earlier, or there is a deviation from the typical eruption sequence, maxillary incisor eruption is considered delayed [2]. The incidence of maxillary central incisor impaction spans between 0.06% and 0.2% [3].

Following the third molar, the upper canines are the next most commonly impacted teeth with an incidence of the same ranging from 1% to 2.5% [4]. Impacted upper canines are frequently addressed in orthodontics. The maxillary cuspid has the most difficult and complex eruption path among any teeth. Initially, it is placed high in the maxilla at the age of three, with its crown directed mesiolingually. While advancing to the occlusal plane, it gradually straightens out until it contacts the distal side of the lateral incisor root. It then continues to erupt in a more vertical direction, ultimately erupting in the oral cavity with a distinctive mesial inclination. This tortuous route makes the canine more prone to impactions [5].

## Case presentation

**Figure 1:**
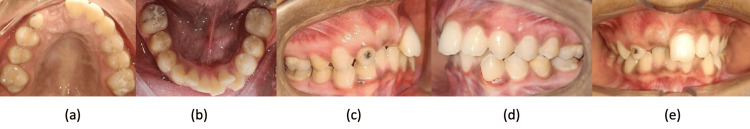
Pre-treatment intra-oral images: (a) maxillary arch, (b) mandibular arch, (c) right molar in occlusion, (d) left molar in occlusion, and (e) anteriors in occlusion Pre-treatment images reveal missing 11, 12, and 13 and crowding in the lower anterior region

**Figure 2:**
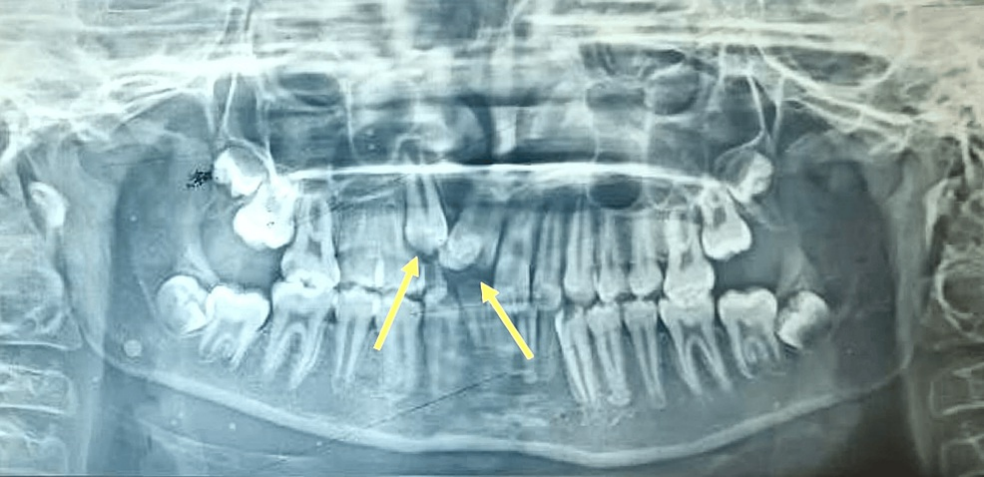
Pre-treatment orthopantomogram (OPG) OPG reveals impacted 11, 12, and 13 along with over-retained 53

**Figure 3:**
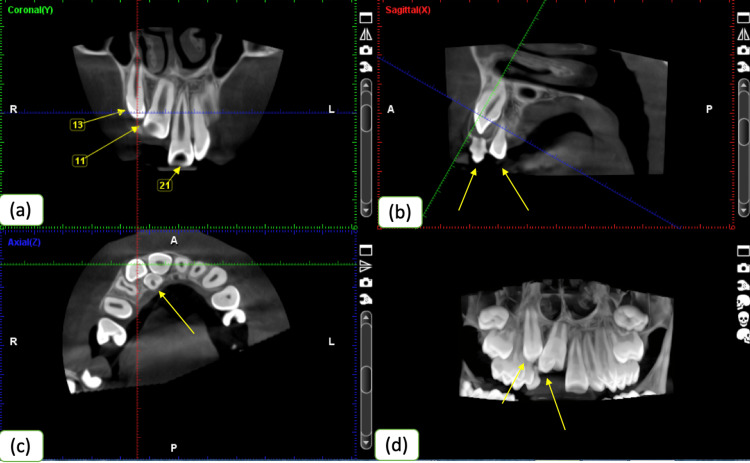
CBCT showing the position of the impacted teeth: (a) coronal section, (b) sagittal section, (c) axial section, and (d) 2D image CBCT, cone-beam computed tomography; 2D, two-dimensional

A male patient, 13 years of age, came with the primary complaint of missing anterior teeth and poor esthetics. Intra-oral examination showed missing 11, 12, and 13, as well as over-retained 53 (Figure 1). Crowding was seen in the lower anterior region along with Angle's class I molar relation on the left side, class II molar relation on the right side, and class I canine relation on the left side intra-orally. The intra-oral findings were confirmed using an orthopantomogram (OPG), which revealed impacted 11, 12, and 13 (Figure 2). A cone-beam computed tomography (CBCT) was taken to confirm the positions of the same (Figure 3).

**Figure 4:**
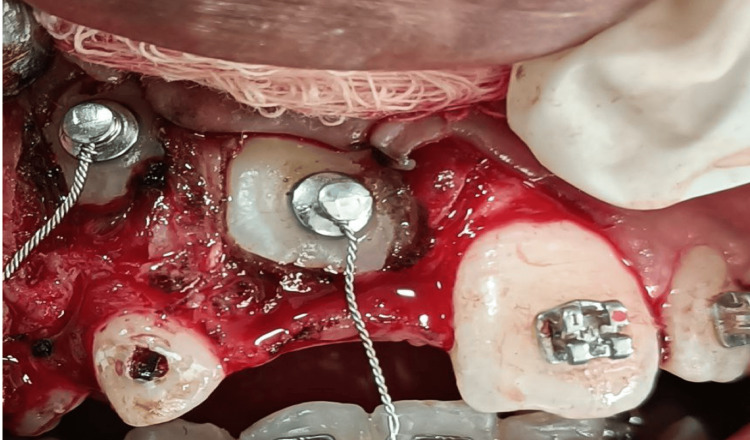
Surgical exposure of impacted 11 and 13

**Figure 5:**
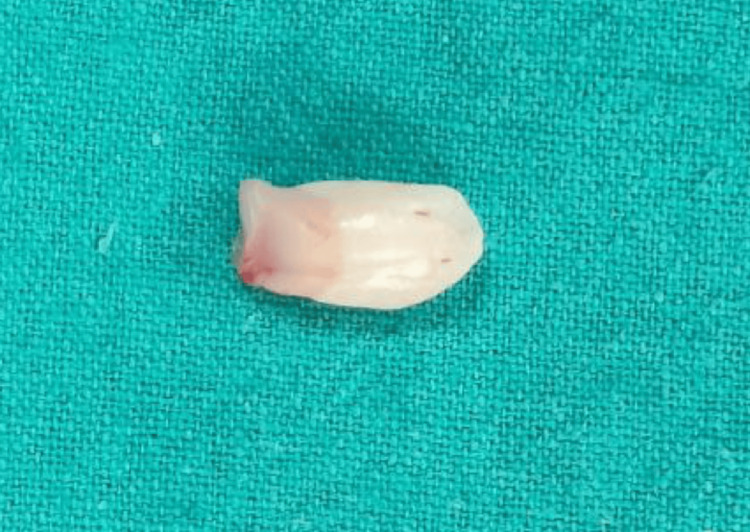
Rudimentary 12 On surgical exposure, 12 was found to be rudimentary and hence extracted

**Figure 6:**
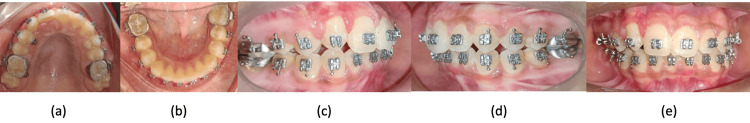
Post-treatment intra-oral images: (a) maxillary arch, (b) mandibular arch, (c) right molar in occlusion, (d) left molar in occlusion, and (e) anteriors in occlusion

**Figure 7:**
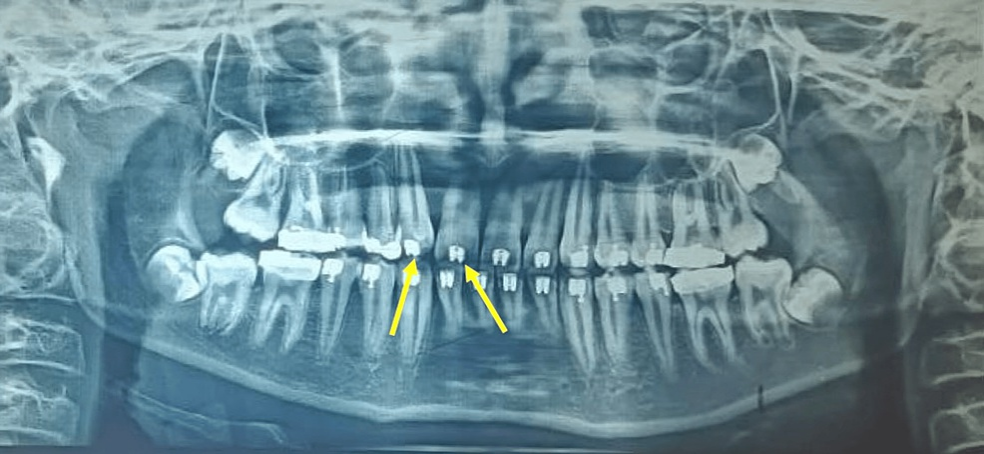
Post-treatment orthopantomogram (OPG) Post-treatment OPG reveals proper alignment of 11 and 13

**Figure 8:**
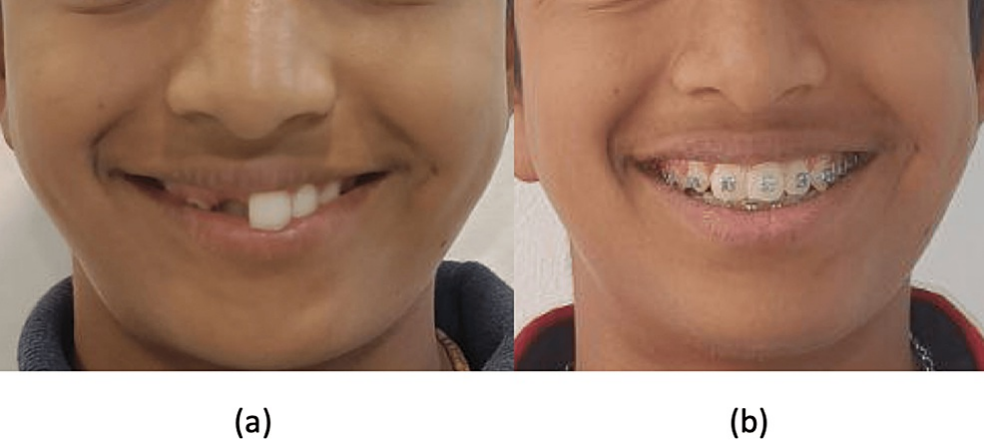
Comparison of pre- and post-treatment frontal smiling

The treatment was started by strapping up the upper and lower arches. Immediately after the initial alignment of the upper arch using 0.014" and 0.016" nickel-titanium (NiTi) wire, the surgical exposure of the central incisor and canine along with the extraction of over-retained 53 was done (Figure 4). Since, after exposure, the lateral incisor was found to be rudimentary, it was extracted (Figure 5). A traction force was applied on the exposed teeth by bonding lingual buttons, which were later aligned by a segmental piggyback NiTi wire. The complete levelling and alignment of both arches were done using NiTi wires followed by stainless steel wires (Figure 6). The post-treatment OPG shows proper alignment of 11 and 13 (Figure 7). The pre- and post-treatment smiling photographs are compared in Figure 8. The entire treatment was completed in 16 months.

## Discussion

Impacted teeth are a common occurrence in orthodontic practice. McBride believes that the lack of eruptive force to permanent teeth to bring them into their natural location in the oral cavity typically occurs due to an imbalance between tooth size and overall arch length. When there is such a discord, the teeth that erupt later in the series are either impacted or diverted from their regular eruption courses [6].

For many years, experts have been interested in the reasons for maxillary permanent canine eruption disturbance and impaction. Compared to all other teeth, the upper canines have the most challenging path of eruption and a long development period. Although the etiology of ectopic canines is unknown, it is most likely multifaceted. Both genetic [7-9] and local factors have been reported to be linked with canine impactions, which occur in a small but considerable proportion of most populations [10-12].

Several therapeutic options have been developed. Alveolar bone loss is to be expected if the affected tooth is extracted. According to Lin et al., alveolar bone resorption is almost inescapable and frequently happens during the first six months post extraction [13]. In such cases, the exposure of the affected tooth surgically followed by orthodontic traction is becoming more popular. A number of studies have indicated that a tooth impacted in the dental arch can be aligned. However, numerous factors may influence whether that tooth can be effectively aligned: (a) the location and orientation of the impacted tooth, (b) the degree of root formation, (c) dilacerations, and (d) the available space for the impacted tooth [14,15].

## Conclusions

In conclusion, the presented case of impacted teeth 11, 12, and 13 highlights the intricate nature of managing multiple impacted teeth and the importance of a thorough treatment plan. This case demonstrates the significance of individualized and coordinated care in dealing with such difficult dental problems. As we navigate the complex nature of dental anomalies, the lessons acquired from this case report serve as a guide for clinicians, emphasizing the importance of adaptability, innovation, and a comprehensive approach to patient care.
